# SenseHunger: Machine Learning Approach to Hunger Detection Using Wearable Sensors

**DOI:** 10.3390/s22207711

**Published:** 2022-10-11

**Authors:** Muhammad Tausif Irshad, Muhammad Adeel Nisar, Xinyu Huang, Jana Hartz, Olaf Flak, Frédéric Li, Philip Gouverneur, Artur Piet, Kerstin M. Oltmanns, Marcin Grzegorzek

**Affiliations:** 1 Institute of Medical Informatics, University of Lübeck, Ratzeburger Allee 160, 23562 Lübeck, Germany; 2 Department of IT, University of the Punjab, Katchery Road, Lahore 54000, Pakistan; 3 Department of Management, Faculty of Law and Social Sciences, Jan Kochanowski University of Kielce, ul. Żeromskiego 5, 25-369 Kielce, Poland; 4 Section of Psychoneurobiology, Center of Brain, Behavior and Metabolism, University of Lübeck, Ratzeburger Allee 160, 23562 Lübeck, Germany; 5 Department of Knowledge Engineering, University of Economics in Katowice, Bogucicka 3, 40-287 Katowice, Poland

**Keywords:** hunger, satiety, physiological signals, non-invasive sensing, multimodal sensing, machine learning, artificial neural network

## Abstract

The perception of hunger and satiety is of great importance to maintaining a healthy body weight and avoiding chronic diseases such as obesity, underweight, or deficiency syndromes due to malnutrition. There are a number of disease patterns, characterized by a chronic loss of this perception. To our best knowledge, hunger and satiety cannot be classified using non-invasive measurements. Aiming to develop an objective classification system, this paper presents a multimodal sensory system using associated signal processing and pattern recognition methods for hunger and satiety detection based on non-invasive monitoring. We used an Empatica E4 smartwatch, a RespiBan wearable device, and JINS MEME smart glasses to capture physiological signals from five healthy normal weight subjects inactively sitting on a chair in a state of hunger and satiety. After pre-processing the signals, we compared different feature extraction approaches, either based on manual feature engineering or deep feature learning. Comparative experiments were carried out to determine the most appropriate sensor channel, device, and classifier to reliably discriminate between hunger and satiety states. Our experiments showed that the most discriminative features come from three specific sensor modalities: Electrodermal Activity (EDA), infrared Thermopile (Tmp), and Blood Volume Pulse (BVP).

## 1. Introduction

Hunger and satiety perception occurs within the hypothalamic areas of the brain, processing a number of endocrine signals coming from peripheral organs such as the stomach, liver, pancreas, intestine, or fat tissue [1]. Differentiating between hunger and satiety is crucial to maintaining stable body weight and preventing malnutrition. Specifically, overweight and obesity are known to be associated with a gradually advanced loss of this perception, leading to overeating, underlying the disease [2]. According to the World Health Organization (WHO), 39% of adults aged 18 years and older were overweight, and 13% were obese in 2016 [3]. So far, common methods to determine hunger and satiety are invasive, i.e., via hormonal analyses from blood samples, or based on self-assessment, such as Visual Analog Scales (VAS) [4,5]. The latter records subjective sensations such as the desire to eat, hunger, satiety, and nausea [6,7] and by nature, underlies several external factors influencing the test results (e.g., stress level, environmental temperature, etc.). In contrast, invasive methods—mostly used in experimental settings—measuring blood concentrations of relevant hormones are not practicable in everyday life. In order to develop a therapeutic device that may assist people to train hunger and satiety perception, objective and non-invasive measurements are necessary.

The detection of hunger and satiety with multimodal physiological sensor signals using supervised machine learning (ML) is a worthy investigation. This is because ML has already shown promising results on physiological sensor signals in a various applications in other fields such as biology, medicine, and psychology [8,9,10,11]. An important step in a ML process is *feature extraction*, which consists of computing some values from the data—referred to as *features*—that are meaningful for the problem to solve. Feature extraction approaches map the data from a high-dimensional space to a low-dimensional one to lower the complexity of the ML problem. There are two main families of feature extraction, namely feature engineering and feature learning. Feature engineering refers to the manual crafting of features, either based on expert knowledge or on simple transformation functions (e.g., arithmetic operators and/or aggregation operators) applied to the sensor signals.

Feature learning, on the other hand, designates the automated learning of features from the data. One of the most popular feature learning approaches nowadays is deep learning that is based on Artificial Neural Networks (ANNs). They work in an end to end fashion and have already shown promising results in a large number of health-related applications [12,13,14,15,16]. ANNs are modeled after their biological counterparts and can be implemented on computers as software applications. The basic elements of ANNs are artificial neurons, which are interconnected in form of layers. Sensor signals are provided to the input layer, and then they move to the output layer via interconnected neurons. An ANN, which consists of more than three layers, i.e., an input layer, an output layer, and several hidden layers, is called a Deep Neural Network (DNN). DNNs can be trained with appropriate data to create a useful model that converts inputs into outputs [17,18].

Developing an objective system to predict hunger and satiety using multimodal sensory signals is a complex task. However, such a problem has not been explored extensively in the past literature. More specifically, all past studies either used invasive sensor modalities or investigated a related but different problem than the recognition of hunger and satiety. In this work, we therefore hypothesize that modern non-invasive wearable sensors can allow us to distinguish hunger and satiety states. We perform an ML study involving the comparison of several state-of-the-art feature extraction and classification approaches. We also investigate various sensor modalities recording physiological data to determine which one(s) contribute the most to this problem.

To summarize, we make the following contributions:

1. We investigate the use of non-invasive multimodal sensors in the context of hunger and satiety detection and develop a state-of-the-art machine learning model, which learns hunger and satiety patterns from multimodal sensors data and classifies them into hunger and satiety classes.
2. We analyze and compare wearable devices and sensor channels to select the most relevant physiological signals for an accurate classification of hunger and satiety data.
3. We perform a comparative analysis of feature extraction approaches and machine learning algorithms to identify the best features in achieving optimal classification results.
4. We also provide a brief review of related approaches.

The rest of the article is structured as follows. Section 2 presents the current state-of-the-art in hunger and satiety detection. Section 3 describes the materials and methods used to analyze multimodal signals for assessing hunger and satiety. Section 4 presents the experimental results. Section 5 provides a discussion, and finally, Section 6 concludes this work.

## 2. Related Work

In recent years, some hunger detection methods have been applied for clinical and behavioral assessments [4,19,20,21,22,23,24,25]. Table 1 lists the sensors and systems used in the reviewed studies.

To the best of our knowledge, physiological signals acquired from multimodal sensors have not yet been used for the prediction of hunger and satiety responses using machine learning. For example, Barajas-Montiel and Reyes-Garcia [25] applied traditional signal processing and pattern classification methods to detect hunger cries, no-hunger cries, pain cries, and no-pain cries from infant acoustic data. Here, the detection of hunger cries and no hunger cries is based on acoustic features in the form of frequencies. The model proposed in this paper [25] is specific to infants and could not be generalized to the young and elderly population to detect hunger and satiety. They did not describe feature learning or the use of wearable physiological sensors for hunger and satiety detection.

Interestingly, Maria and Jeyaseelan [23] used audio signals generated by the stomach to identify growls that can describe hunger well. The synthetic audio signals were recorded using mobile phones and pre-processed using smoothing methods and median filtering. Spectral features were calculated to classify the signals into growls and burps.

Krishnan et al. [4] used ANN to model the feelings of hunger and satiety after food intake. They trained their model with a dataset relating concentration–time courses of plasma satiety hormones to VAS assessments. The proposed model successfully predicted VAS responses from the dataset of satiety hormones obtained in experiments with different food compositions. They also revealed that the predicted VAS responses for the test data separated the satiety effects of highly satiating foods from less satiating foods, for both oral and ileal infusion. However, their approach is time-consuming and invasive because they used plasma hormone levels, which are not easy to obtain compared to physiological signals detected by smart sensor devices.

Bellmann et al. [19] claimed that human clinical trials are time-consuming and costly. Therefore, they developed a gastrointestinal model in conjunction with ANN to predict feelings of hunger and satiety after the ingestion of different meals. They trained their model with a series of training datasets to create a prediction set and link the model measurements to VAS scores for hunger and satiety. Although gastrointestinal-based modeling is still in its infancy, it is evident that the development of machine learning approaches has the potential to transform such models into powerful predictive tools, which can predict physiological responses to food. However, the acquisition of physiological responses by miniaturized sensors is state-of-the-art.

Rahman et al. [20] proposed that predicting eating events can enable users to adopt better eating behaviors. As a consequence, they used a set of sensor devices to record physical activity, location, heart rate, electrodermal activity, skin temperature, and calories ingested while eight users were eating. They extracted 158 window-level features, followed by correlation-based feature selection (CFS), and trained a classifier to predict the about-to-eat event. Time until the next eating event was predicted using regression analysis. However, the use of motion sensors such as accelerometers and gyroscopes is questionable for the “time until the next eating” event. Additionally, they did not provide any comparison between sensor modalities to determine the best optimal device.

Al-Zubaidi et al. [21] investigated the influence of hunger and satiety on resting-state functional magnetic resonance imaging (rs-fMRI) using connectivity models, i.e., local connectivity, global connectivity, and the amplitude of rs-fMRI signals. They extracted the connectivity parameters of ninety brain regions for each model and used the sequential forward sliding selection strategy in conjunction with a linear support vector machine classifier to determine which connectivity model best discriminated between metabolic states (hunger vs. satiety). They claimed that the amplitude of the rs-fMRI signals, with a classification accuracy of 81%, is slightly more accurate than the local and global connectivity models in detecting changes in the resting state of the brain during hunger and satiety. However, they did not show results with the state-of-the-art supervised feature learning approach.

Gogate and Bakal [24] presented a hunger- and stress-monitoring system using galvanic skin response data from 35 patients using proprietary data processing and classification techniques. They claimed an overall accuracy of the system of 86.6%. However, they did neither specify a method for data processing and feature extraction, nor did they use classical or modern classification methods.

Lakshmi et al. [22], proposed a method to detect hunger specifically in physically disabled people. The main goal was to communicate using the brain’s thoughts without muscle control, specifically for severely paralyzed people with a non-invasive approach to make the task less complex and more convenient. In this approach, a single-channel electrode was placed on a person’s scalp to detect human sensations of hunger, thirst, and toilet using images placed in front of it. The final result was obtained by analyzing the person’s attention level. The attention levels of each image were compared to the corresponding image in MATLAB, and the resulting attention level value was obtained.

In general, there are very few studies [4,19,20,21,22,23,24,25] on the subject that we investigate. However, each of them has some limitations; for example, the data collection method used by Krishnan et al. [4] was invasive, and the results of Bellmann et al. [19] were based on gastrointestinal models. Rahman et al. [20], used motion sensors for the “time until the next eating” event, which is questionable. Maria and Jeyaseelan [23], and Barajas-Montiel and Reyes-Garcia [25] used microphones to record the data, which can trigger a privacy risk. The authors in [21,22,24] used hand-crafted features, while feature learning can perform as well or better than state-of-the-art [26]. To-date, no automated system for detecting hunger and satiety using multimodal physiological signals has been evaluated, nor is there a public dataset.

## 3. Materials and Methods

**Figure 1:**
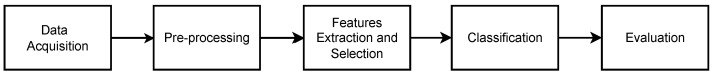
Standard approach to developing machine learning and pattern recognition systems. Each step should be optimized in parallel to achieve the best performance.

In this section, we present the aspects of the sensor modalities accumulated for data acquisition, the process of data acquisition, and discuss the experimental settings. The entire process from data acquisition to analysis consists of a series of steps as shown in Figure 1, which has been extensively described in the past literature [9,27].

### 3.1. Dataset Acquisition

**Figure 2:**
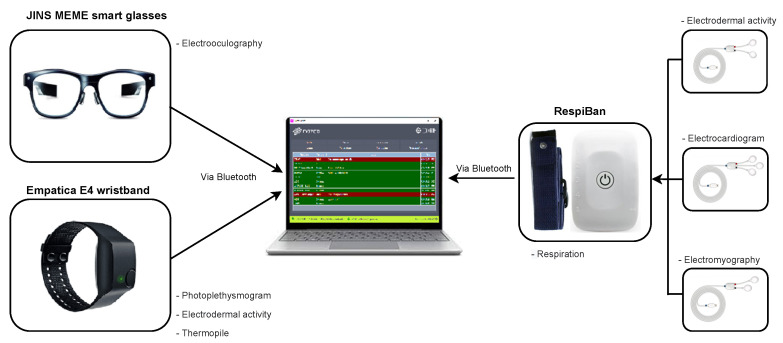
The SenseHunger system uses three sensory devices, namely, JINS MEME smart glasses, Empatica E4 wristband, and RespiBan. The Electrodermal activity (EDA), Electrocardiogram (ECG), and Electromyography (EMG) electrodes are plugged into the RespiBan device. Datasets from all devices are sent to the laptop for storage using a Bluetooth connection.

The hardware configuration of our proposed sense-hunger system is shown in Figure 2. We used the following wearable devices and sensor modalities to collect physiological hunger and satiety signals from five healthy individuals:

1. RespiBan (Plux Wireless Biosignals S. A., Lisboa, Portugal) [28]: Subjects wear the respiration belt on the chest, at the level of the thorax, with the electrode connectors facing forward. It contains the Respiration (Resp) sensor and also provides the possibility for connecting to other sensors such as Electrodermal activity (EDA), Electrocardiography (ECG), and Electromyography (EMG), as shown in Figure 2. The description of these sensors is as follows:
  - Resp: This sensor measures the respiration rate. It detects chest or abdominal expansion/contraction, and outputs a respiration signal. It is usually worn using a comfortable and flexible length-adjustable belt. It is sampled at 475 Hz.
  - EDA [29]: EDA of RespiBan (Eda_RB) consists of two electrodes placed on the front, in the middle of the index finger, and in the middle of the middle finger of subject’s non-dominant hand. This sensor measures the galvanic skin response, i.e., the change in electrical conductivity of skin in response to sweat secretion. It is also sampled at 475 Hz.
  - ECG [30]: It consists of three electrodes placed on the subject’s right upper pectoral, left upper pectoral, and at the left bottom thoracic cage. This sensor records the electrical impulses through the heart muscle, and it can also be used to provide information on the heart’s response to physical exertion. It is also sampled at 475 Hz.
  - EMG [31]: This sensor is used to assess the electrical activity associated with muscle contractions and respective nerve cells, which control them. It is placed on the subject’s abdomen above the belly button and is also sampled at 475 Hz.
2. Empatica E4 wristband (Emaptica Inc., Cambridge MA, USA) [32]: It contains photoplethysmogram (PPG), infrared thermopile (Tmp), and EDA sensors that allow measurements of sympathetic nervous system activity and heart rate (HR) variability. The description of these sensors is as follows:
  - PPG: This sensor measures blood volume pulse (BVP), which can be used to derive HR and inter-beat interval (IBI). It is sampled at 1 Hz.
  - Tmp: This sensor records skin temperature. It is sampled at 5 Hz.
  - EDA: EDA of Empatica E4 (Eda_E4) wristband measures the galvanic skin response, which is the change in the electrical conductivity of the skin in response to sweat secretion. It is sampled at 5 Hz.
3. JINS MEME smart glasses (Jins Inc., Tokyo, Japan) [33]: They can track not only where we look, but how often we blink and even whether we are about to relax or fall asleep. It uses electrooculography (EOG) electrodes placed in three locations on the frame. These electrodes can track blink duration and eye movements in different directions. It is sampled at 20 Hz.

The data collection of hunger and satiety activities involved five healthy volunteers whose demographic information is provided in Appendix C. Subjects were asked not to eat anything for 16 h before data collection. However, drinking water was allowed. Data collection for each subject was divided into two phases, namely, the hunger and the satiety phase. In the hunger phase, data collection lasted for 5 min, using the sensory devices shown in Figure 2. After eating, the process was resumed for the satiety phase, which lasted for 30 min.

### 3.2. Pre-Processing

State-of-the-art machine learning (ML) algorithms can certainly derive knowledge from raw sensor data. However, their output generally depends on the quality of the datasets they are working with. If data are insufficient or contain extraneous and irrelevant information, ML algorithms may produce less accurate and less understandable results or discover nothing useful at all. Therefore, pre-processing of the data is an important step in the process of ML. The pre-processing step is necessary for solving various types of problems influencing data such as noise, redundancy, missing values, etc. [34]. In the first step, datasets from all sensor channels (as shown in Figure 2) are synchronized, resampled to a frequency of 100 Hz, and linearly interpolated to ensure that the channels shared a common repetition.

Based on our preliminary experiments, we segmented the data of each sensor channel using a Sliding Window Segmentation (SWS) in the following three settings with an overlapping window, to select the optimal setting: In the first setting, the length *T* and sliding stride (step size) Δ*S* of a time window are set to 10 and 5 s, respectively. The second setting is defined by length *T* = 30 s and sliding step Δ*S* = 15 s, while in the third setting, the length *T* and the sliding step Δ*S* of a time window are set to 60 and 30 s, respectively. The experimental results with the mentioned window sizes and step sizes are presented in Section 4.

### 3.3. Feature Extraction and Selection

In a linear or nonlinear fashion, feature extraction approaches model the data from a high-dimensional space into a reduced dimensional space. In this study, we used two approaches to extract features, namely the hand-crafted features and automated feature learning.

Hand-crafted Features: We used 18 hand-crafted features [9,35] consisting of the statistical and frequency-related values of the input signals. These features are listed in Table 2. All features were computed independently for each axis of each sensor channel, following the suggestions of Cook and Krishnan [36]. They were subsequently concatenated to obtain a feature vector of size 18 × sensor (S). To remove the effects of discrepancies between the values of each feature, min-max normalization was performed for each feature to project its values into the interval [0,1]. The normalization constants calculated on the training set were again used to calculate the features in the test set.

We applied feature selection on the features we manually computed to remove useless or redundant ones, and to decrease the complexity of our classification model. This can improve the performance of a model and determine the interdependence between features and class labels [36]. A common approach for feature selection is feature ranking, which quantifies the ability of the feature to predict the desired class. A Random Forest (RF) was used to select the most important hand-crafted features [37]. It is a tree-based learner that generally grows by applying the classification and regression tree method (CART) [38], where binary splits recursively partition the tree into homogeneous or nearly homogeneous terminal nodes. After a fair split, the data is moved from the root tree node to the child nodes, improving the homogeneity of the child nodes relative to the parent node [39]. Typically RF consists of a set of hundreds of trees, where each tree is grown using a sample of the dataset.

In RF, trees are generally grown non-deterministically using a two-step randomization procedure. Apart from the randomization applied by growing the tree using a sample of the primary data, a subsequent level of randomization is set at the node level as the tree grows. The objective of this two-step randomization is to decorrelate the trees, so that RF ensemble has low variance. Features ranked by RF are based on the quality of the purity improvement (which is the fraction of data items that belong to the class) of the node. Given a node n and the estimated class probabilities p(k|n)k=1,…Q. The Gini index can be defined by using the following equation [40].

(1) $$G(n)=1-\sum_{k=1}^{Q}{p(k|n)}^{2}$$

In Equation (1), *Q* is the total number of classes. In order to obtain the Gini index-based measure at each node, the Gini index decline is calculated for the variable used for partitioning. The Gini index-based measure of variable importance is then obtained by the average drop in the Gini index. For the comparison of manual feature selection approaches, see Appendix A.

Feature Learning: Feature learning involves learning features from labeled input data in an automated way without any human input. Feature learning has become increasingly popular over the past years with the popularization of ANNs and DNNs. During training, they are fed with raw input data to learn a mapping against each class in an end to end fashion. ANN and DNN models have been shown to perform well on various tasks (e.g., image classification [41], activity recognition [9,42], and sleep stage classification [8]). However, training such models can be challenging as it is computationally more expensive than training traditional models. Moreover, finding optimal architectures is a non-trivial process.

**Figure 3:**
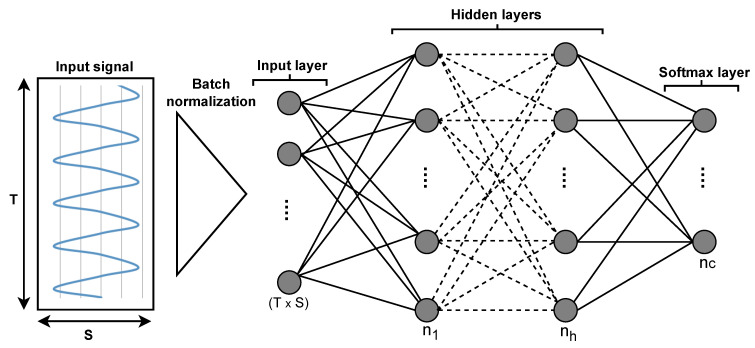
Illustration of a MLP where different sensor channels are converted into a (T×S) dimensional vector, which is passed to the different hidden layers (h) and output classes (c) as defined by the softmax layer.

In the past, Multi-Layer Perceptrons (MLPs) [43] and Convolutional Neural Networks (CNNs) [44] have been used for various tasks. MLPs represent the most primitive type of ANN. In order to process 2D sensor data with its sensor axis (S) and time (T), the input data are first normalized using the batch-normalization layer [45], and then passed to fully connected layers that expect 1D input. A syntactic example of the MLP architecture can be seen in Figure 3.

**Figure 4:**
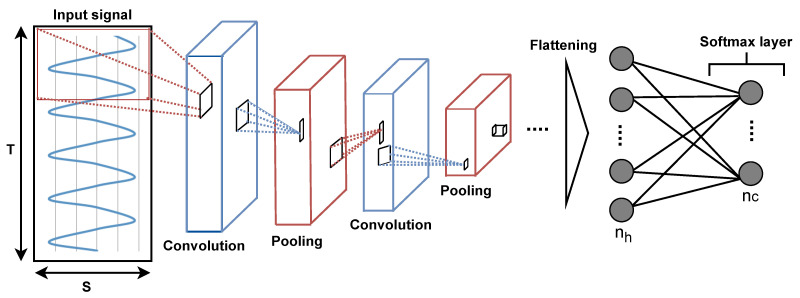
Illustration of a Convolutional Neural Network (CNN) model with convolutional layers, pooling layers, h dense layers, and c output classes represented by a softmax layer. Input data are processed by convolutional layers and pooling layers, and are passed to dense layers after extraction of profound features.

In CNN architectures, the convolutional layers are the main building blocks normally used to perform convolutional operations between one or several convolutional filters (or kernels) learned during the training phase and the layer input. The convolution operation can be applied by sliding the convolution kernels over the input data. In this study, raw sensor data are given as 3D input (S× T× 1) to the CNN model for processing. After a series of convolutional and pooling layers, the output of the last convolutional layer is usually smoothed into a 1D vector and fed into the softmax layer. The Rectified Linear Unit (ReLU) is the most commonly used activation function for convolutional layers. It is also common to add multiple dense layers of a multilayer perceptron to the CNN architecture for classification problems. In that case, a softmax activation function is usually used to connect the aftermost dense layer to the output layer. An example of a CNN model can be seen in Figure 4.

In initial experiments (whose results are reported in Appendix B), various configurations for the window size (*T*), step size (Δ*S*), and learning rate (*lr*) parameters were examined. It was found that *T* = 60 s, Δ*S* = 5 s, and *lr* = $10^{-4}$ yielded the best performances. Therefore, each sensor channel information was segmented into parts, resulting in data frames of the form (N×S×1), where *N* is the number of segments, or more precisely, (6000×7×1) for each class.

The purpose of this study was to test the use of feature learning methods with a dual objective. The primary goal was to analyze the quality of MLP and CNN in automatically extracting features with different hyperparameters. The secondary objective was to examine and compare the results of human-generated features and automatic feature extraction. The results of classifying hunger and satiety using the above mentioned approaches are presented in the experimental results section.

### 3.4. Classification

To provide a comparison between hand-crafted features and automatically learned features, we used two types of classification approaches. Traditional classifiers such as support vector machine (SVM), decision tree (DT), and RF were trained and tested on hand-crafted features, and ANN-based models such as MLP and CNN with softmax layers were applied to classify the automatically learned features into hunger vs. satiety classes. The description of these methods are as follows:

1. SVM: In pattern recognition, SVM is a supervised learning algorithm, which can be used for classification and regression tasks. Its robust performance on noisy and sparse data makes it a good choice for a variety of applications [42]. In a classification task, the SVM separates the labeled training data with a maximum margin hyperplane. Test data are then mapped to the same space to predict a class label. SVM can also efficiently map high-dimensional data to a high-dimensional dimension feature space to perform nonlinear classification [46].
2. DT: This is an approach to classification or regression analysis, in which a decision tree is constructed by recursively partitioning the feature space of the training set into smaller and smaller subsets. The final consequence is a tree with decision and leaf nodes. DT aims to find a set of decision rules that instinctively divide the feature space to build a instructive and robust classification model. A decision node has binary or multiple branches. A leaf node indicates a class or outcome. The top decision node in a tree points to the best predictor, which is called the root node [47].
3. RF: This is a popular ensemble learning method used for various types of classification problems such as activity recognition [35], where multiple DTs are created at training time [48,49,50,51,52]. In RF, each tree casts a unit vote by assigning each input to the most likely class label. RF is fast, robust to noise, and an effective ensemble, which can be used to identify nonlinear patterns in datasets. It can handle both numeric and categorical data. The biggest advantage of RF compared to DT is that it is significantly more resilient to overfitting [53].

### 3.5. Evaluation

The selection of the evaluation metric is very important and application-dependent, because an inadequately defined metric may lead to incorrect conclusions [54]. For this reason, the evaluation metrics were designed to be consistent with the state-of-the-art work in this field, and to facilitate comparison. It is worth mentioning that in all experiments of this work, cross-validation was used according to the Leave-One-Subject-Out (LOSO) protocol, in which each subject’s data are used once as the test set, whereas the remaining data constitute the training set. In general, the overall performance is the average of the results gained for each tested subject. The LOSO cross-validation procedure guarantees that all models are tested on unknown subjects, which allows a realistic evaluation of the classification algorithms used in de-factor applications.

For the classification performance of the different models tested, we used accuracy assessed by the ratio of true predictions (i.e., true positive (*t_p_*), true negative (*t_n_*)) to all entries (i.e., true positive (*t_p_*), true negative (*t_n_*), false positive (*f_p_*), false negative (*f_n_*)) [55], as shown in Equation (2):

(2) $$\mathrm{Accuracy}=\frac{t_{p}+t_{n}}{t_{p}+t_{n}+f_{p}+f_{n}}$$

In addition to the accuracy, we used the averaged F1 (AF1) score (short for macro-averaged F1 score), which treats all classes equally and can be used to evaluate the class imbalance problem (as shown in Equation (6)). It can be defined by using Precision (Equation (3)), Recall (Equation (4)), and F1 score (Equation (5)) [55,56].

(3) $$\mathrm{Precision}=\frac{t_{p}}{t_{p}+f_{p}}$$

(4) $$\mathrm{Recall}=\frac{t_{p}}{t_{p}+f_{n}}$$

The F1 score combines the precision and recall into a single metric by taking its harmonic mean, as shown in Equation (5):

(5) $$\mathrm{F1 score}=\frac{2\times \mathrm{Precision}\times \mathrm{Recall}}{\mathrm{Precision}+\mathrm{Recall}}$$

In our experiments, the AF1 score is given, which is the average of the F1 scores of all classes:

(6) $$\mathrm{AF1 score}=\frac{1}{c}\sum_{i=1}^{c}{\mathrm{F1 score}}_{i}$$

In Equation (6), c represents the no. of classes and $F1\;{\mathrm{score}}_{i}$ represents the F1 score for the *i*th class.

## 4. Experimental Results

In our study, all algorithms and models were implemented using Python 3.9. For the algorithms SVM, DT, and RF, and the deep learning models MLP and CNN, the libraries sklearn and Keras with Tensorflow 2.2.0 backend were used. Adaptive Moment Estimation (ADAM) [57] was chosen as the optimizer for our deep learning model with an initial learning rate of $10^{-4}$, and trained with 50 epochs at a batch size of 32. The categorical cross entropy was used as the loss function for the deep learning models. Since no automated method for the optimization of DNN hyper-parameters has been found so far, trial-and-error was used to obtain the best hyper-parameters for the DNNs we tested in our study. The configurations we tested are provided in Appendix B. The hyper-parameter values that were used in our experiments are provided in Table 3 and Table 4 for MLP and CNN, respectively. It is worth mentioning that we decided not to report the result of a single LOSO cross-validation, but the average results obtained after performing it five times.

Preliminary experiments with all hand-crafted features (i.e., without feature selection), and SVM, DT, and RF classifiers were carried out to determine the best segmentation parameters. The results of these experiments are shown in Table 5. It can be seen that the best performing configuration is obtain when using RF with T = 60 s and ΔS = 30 s, and largely outperforms the others that were tested. We therefore selected these segmentation parameters and classifier for the rest of our studies. However, the overall classification results remain mediocre, with a AF1 score of around 60%.

**Figure 5:**
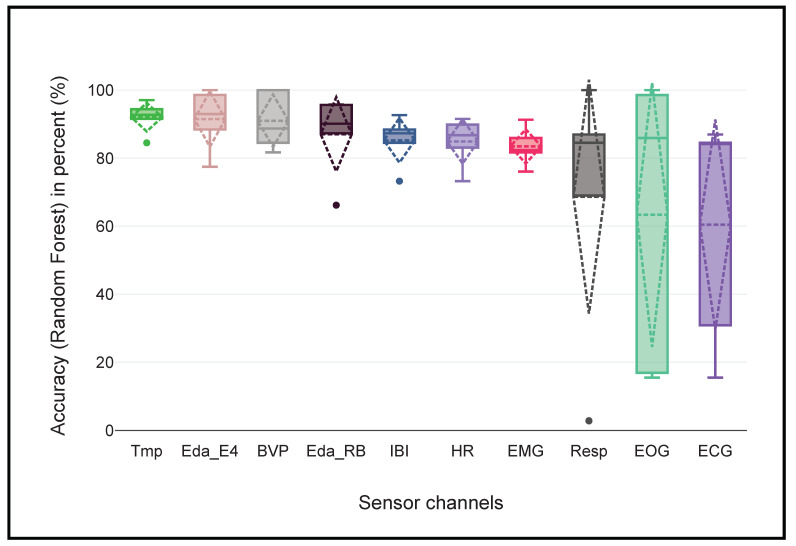
Importance of each sensor channel in recognizing hunger and satiety.

To improve the initial classification results and verify the potential of each sensor channel, experiments were also conducted with each sensor channel separately. We monitored the classification accuracies of each sensor channel after the LOSO cross-validation to determine its relevance in detecting hunger and satiety. Figure 5 shows the boxplot, mean, and standard deviation (in dotted lines) of the obtained accuracies.

The standard deviations of Resp, ECG, and EOG are higher compared to the other sensors. The results in Table 6 show that these sensors are the least significant because their accuracy is less than 70%, and there is a very large variance among the different subjects. Therefore, we decided to exclude the Resp, ECG, and EOG sensors data for further experiments. Moreover, the literature also confirms the importance of Tmp, BVP, and EDA (Eda_E4 and Eda_RB) signals in the detection of hunger. For example, the research of Mandryk and Klarkowski [58] reveals that BVP increases in response to hunger and decreases in response to relaxation, He et al. [59] identifies changes in Tmp, EDA, and HR values following the ingestion of food. The authors in [24] had already used EDA for hunger detection. Furthermore, IBI and HR are directly related to BVP, since they are derived from it.

Further experiments were performed with the best 18, 54, 72, 90, and 108 features of the selected sensor channels (i.e., excluding Resp, ECG, and EOG), ranked by their increasing Gini impurity scores. With the best 18 features, an Acc of 93.43% and an AF1 score of 87.86% were obtained, as shown in Table 7.

The results of our experiments shows that the best results could be obtained with just 18 hand-crafted features based on the FIR (as shown in Table 7). Moreover, there is not much difference in the classification results of the best 54, 72, 90, and 108 features. Furthermore, the results with 18 hand-crafted features are notably better than the results that were obtained using all sensors (see Table 5). It could be concluded that Resp, ECG, and EOG are the least informative sensors in this case, while BVP, Eda_E4, Tmp, HR, Eda_RB, and EMG are the most informative sensors and could be used to detect hunger and satiety.

**Figure 6:**
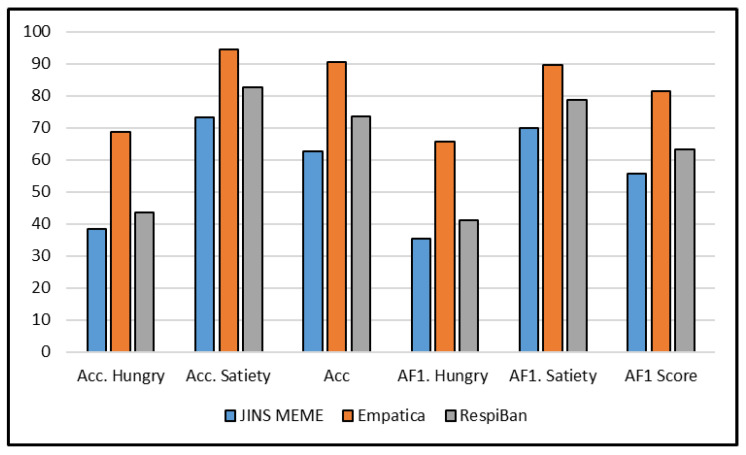
Comparison of sensor devices on the basis of accuracy (Acc) and averaged macro F1 score (AF1) for hungry and satiety classes. Empatica: Empatica E4 wristband; JIMS MEME: JINS MEME smart glasses; RespiBan: RespiBan professional device, including ECG, EMG, and EDA sensors.

To determine the relative relevance of each wearable device (i.e., Empatica E4 wristband, JINS MEME smart glasses, and RespiBan professional, with ECG, EMG, and EDA sensors) in detecting hunger and satiety, further experiments were also conducted with the RF classifier. Figure 6 shows the results of each device using the best 18 features in each case. Our experimental results show that Empatica appears to be the best wearable device, outperforms the other devices, and might be used as the only wearable device for monitoring hunger and satiety.

To provide a comparison between feature engineering and feature learning approaches on our dataset, the experiments were also performed using CNN and MLP. With the CNN, an Acc of 82.90% and an AF1 score of 82.54% were obtained, as shown in Table 8. The segmentation technique mentioned above was not adequate for training a deep learning model. Therefore, we devised another segmentation technique using a window size of 60 seconds and a step size of 5 s for deep learning-based models.

## 5. Discussion

**Figure 7:**
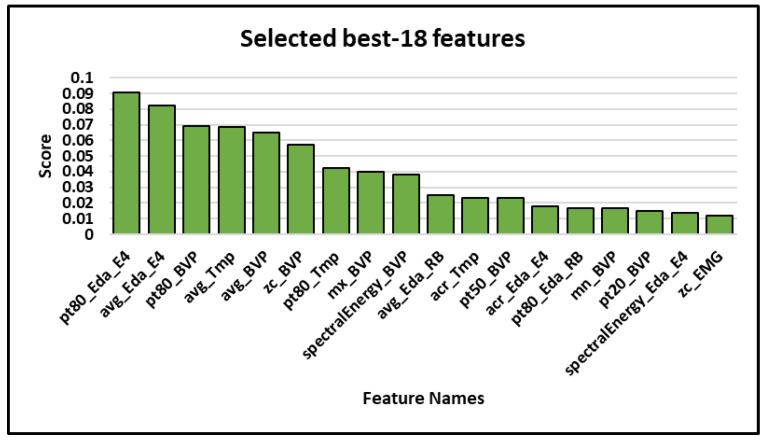
The overall 18 best features. Note: pt80: 80th percentile; avg: average; zc: zero crossings; mx: maximum; acr: auto-correlation; pt50: 50th percentile; mn: minimum; pt20: 20th percentile; BVP: Blood Volume Pulse; HR: Heart Rate; Tmp: Temperature; Eda: Electrodermal activity; RB: Respiration belt; E4: Empatica E4.

The following points provide a detailed discussion of the aforementioned results:

- One of the main objective of this paper was to develop a machine learning approach to classify hunger and satiety using wearable sensors. Therefore, we used wearable devices like the Empatica E4 wristband, JINS MEME smart glasses, and RespiBan professional with miniaturized sensors that provided sufficient quality data and that could capture physiological signals related to the perception of hunger and satiety in patients or people with occupational constraints, as opposed to invasive [4], gastrointestinal model [19], fMRI-based data [21], and gastric tone signals [23]. Our proposed non-invasive multimodal system with carefully selected sensor channels outperformed previous approaches with an accuracy of 93.43% and an average F1 score of 87.86%.
- Each classification algorithm is based on different mathematical models [60], and may produce different results for the same dataset. In order to obtain highly accurate results and to select the best classifier for further experiments, we not only conducted experiments with different classifiers, but also with different window sizes and step sizes. It was found that the RF classifier was best suited for hunger and satiety detection using hand-crafted features, and it outperformed the DT and SVM classifiers in each scenario. It was also observed that the window size of 60 s and the step size of 30 were significant for each classifier.
- In the past, deep learning-based approaches have shown promising results in a variety of application domains such as biology, medicine, and psychology [8,12,13,14,15,42,61]. However, they are computationally expensive and also require a large number of training samples [62] to build successful models compared to traditional approaches using hand-crafted features. To compare the results of feature learning and feature engineering, we also computed 18 features independently for each axis of each sensor channel. They were subsequently concatenated to obtain a feature vector of the size of 18 × sensor (S) axis. It was found that well-engineered features can perform better than deep learning approaches in the case of a limited number of training samples.
- In this study, we used feature importance ranking (FIR), which measures the contribution of each input feature to the performance of the model. It turned out that the most accurate results can be obtained only with the best 18 hand-crafted features (as shown in Table 7) and the addition of other irrelevant and redundant features can introduce noise into the data, which can reduce the performance of a classifier. It can be pointed out that the top five features come exclusively from three different sensor channels (Eda_E4, BVP, and Tmp) and are either computing the mean or the 80th percentile of the data values. Percentile 80 provides an approximation of the maximum value in a data segment that is less sensitive to noise or outliers than the actual maximum computation. This would indicate that the average and upper data values in Eda_E4, BVP, and Tmp are of high importance to distinguish between hunger and satiety. This feature selection also validates our previous results to identify the importance of each sensor channel (Table 6), and seem to confirm findings from the literature that showed these sensor channels to be relevant in detecting hunger and satiety [24,58,59] (c.f. Figure 5). The overall selected best features can be seen in Figure 7.
- Long-term monitoring with a large number of wearable sensors may be uncomfortable for users [63]. Therefore, eliminating irrelevant sensors can decrease the degree of discomfort and improve the robustness of the classification system by reducing the dimensionality and also save a lot of money [64]. In this work, we compared not only all sensors, but also wearable devices, to determine the most suitable sensors and wearable device for hunger and satiety detection. It was found that PPG (BVP, IBI, and HR), EDA (Empatica E4 and RespiBan), Tmp, and EMG were the appropriate sensor modalities for this study, and Resp, ECG, and EOG were the least appropriate. We also found that the Empatica E4 wristband was the most suitable device compared to the other devices.

## 6. Conclusions

In this paper, we introduced an objective and non-invasive machine learning model to detect hunger and satiety using physiological sensor data. Our proposed multimodal system enables the detection of hunger and satiety with an accuracy of 93.43%, and an average F1 score of 87.86% in LOSO configuration. The results of this study lead to the following conclusions: firstly, state-of-the-art wearable sensors provide good quality physiological data on hunger and satiety, and could be used to build a non-invasive and objective system. Furthermore, deep learning architectures do not necessarily perform well, especially when we have a limited number of training samples. In addition, feature selection could help to remove unnecessary and redundant features that lead to noise, which in turn leads to better results. Finally, the experiments of this study showed that the most discriminative features come from three specific sensor modalities: Electrodermal Activity (EDA), infrared Thermopile (Tmp), and Blood Volume Pulse (BVP). These sensors are part of the Empatica E4 wristband, which is the most influential device in this study and can be used as a standalone device. In order to learn more about the perception of hunger and satiety, further experiments with long-term hunger and satiety data are needed, which will not only help to train deep learning models well, but also further divide hunger and satiety into sub-classes to gain further insight, which is part of our future work.

## Figures and Tables

**Table 1:** Sensors and systems for the assessment of hunger in the literature.

Study	Sensors/System	Dataset Information	Features	Detection
Barajas-Montiel and Reyes-Garcia [25]	Microphone	1627—samples of hunger and pain cries (acoustic data of infants)	Acoustic features by means of frequencies	Hunger cry, no-hunger cry, pain cry and no-pain cry
Krishnan et al. [4]	VAS	13—subjects plasma concentrations of satiety hormones from blood samples	Feature learning (ANN)	VAS responses from satiety hormone values
Bellmann et al. [19]	In vitro gastrointestinal model	Gastric viscosity and intestinal digestion from tiny-TIMagc	-	Fullness vs. Hunger
Rahman et al. [20]	Microsoft Band, Affectiva Q sensor, Microphone	8—subjects (3 female, 5 male) from 26 to 54 years	Statistical features	Time until the next eating event, and about-to-eat
Al-Zubaidi et al. [21]	fMRI	24—male subjects from 20 to 30 years (fMRI data)	3—features (DC, ReHo and fALFF)	Neuronal resting state alterations changes during hunger and satiety
Lakshmi et al. [22]	EEG	EEG signals	-	Hunger, thirst, EEG signals sensations
Maria and Jeyaseelan [23]	Microphone	Synthetically collected audio signals through mobile phones	SF, CDF and GCC	Growling vs. Burp sound
Gogate and Bakal [24]	EDA	35—patients ( 20 of them used as control group )	-	Hunger vs. Stress

VAS: Visual analog scales; ANN: Artificial neural network; fMRI: Functional magnetic resonance imaging; DC: Degree of centrality; ReHo: Regional homogeneity; fALFF: Fractional amplitude of low-frequency fluctuations; EEG: Electroencephalography; SF: Spectral features; CDF: Cepstral domain features; GCC: Gammatone cepstral coefficients; EDA: Electrodermal activity; tiny-TIMagc: In vitro gastrointestinal model.

**Table 2:** Hand-crafted features calculated independently for each sensor channel.

Hand-Crafted Features	
Maximum	Minimum
Average	Standard deviation
Zero-crossing	Percentile 20
Percentile 50	Percentile 80
Interquartile	Skewness
Kurtosis	Auto-correlation
First-order mean	Second-order mean
Norm of the first-order mean	Norm of the second-order mean
Spectral energy	Spectral entropy

**Table 3:** MLP architecture with learning rate set to $10^{-4}$.

Layer Name	Neurons/Dropout Rate	Activation
Dense	64	ReLU
Batch Norm	-	-
Dense	16	ReLU
Dropout	0.5	-
Flatten	-	-
Dense	8	ReLU
Dropout	0.5	-
Dense	2	Softmax

**Table 4:** CNN architecture with a fixed dropout rate of 0.5 and learning rate of $10^{-4}$.

Layer Name	No. Kernels (Units)	Kernel (Pool) Size	Stride	Activation
Convolutional	64	(1,1)	(1,1)	ReLU
Batch Norm	-	-	-	-
Convolutional	32	(1,1)	(1,1)	ReLU
Convolutional	16	(1,1)	(1,1)	ReLU
Flatten	-	-	-	-
Dense	2	-	-	Softmax

**Table 5:** Results of binary classification of hunger and satiety.

Classifier	Win Size (*T*)	Step Size (Δ*S*)	Acc. Hungry	Acc. Satiety	Acc	AF1 Score
SVM	10	05	20.90	70.37	56.89	45.63
DT	10	05	27.94	70.40	58.04	49.17
RF	10	05	30.97	71.75	59.90	51.36
SVM	30	15	21.61	68.86	55.43	45.24
DT	30	15	21.93	71.54	58.29	46.73
RF	30	15	**38.59**	73.23	62.71	55.91
SVM	60	30	13.19	69.50	55.00	41.34
DT	60	30	18.44	79.43	67.14	48.93
**RF**	**60**	**30**	36.36	**82.05**	**72.00**	**59.21**

DT: Decision tree classifier; RF: Random forest classifier; SVM: Support vector machine classifier; Acc: Accuracy; AF1 Score: Averaged macro F1 score.

**Table 6:** Hunger and satiety classification results on each sensor channel using RF classifier.

Sensor	Acc. Hungry	Acc. Satiety	Acc	AF1 Score
Tmp	73.08	95.30	92.00	84.19
Eda_E4	70.59	94.98	91.43	82.79
BVP	67.35	94.68	90.86	81.02
Eda_RB	62.18	92.25	87.14	77.22
IBI	43.48	91.95	85.14	67.46
HR	40.45	91.33	84.86	65.89
EMG	30.95	90.58	83.43	60.77
Resp	29.30	79.56	68.29	54.43
EOG	39.25	73.25	62.86	56.25
ECG	21.59	73.66	60.57	47.63

RF: Random forest classifier; Acc: Accuracy; BVP: Blood volume pulse; Eda_E4: Electrodermal activity sensor of empatica E4 wristband; Tmp: Thermopile; IBI: Inter-beat interval; HR: Heart rate; Resp: Respiratory; Eda_RB: Electrodermal activity sensor of RespiBan; ECG: Electrocardiogram; EMG: Electromyography; EOG: Electrooculography. Note: For these experiments, we used a window size of 60 s and a step size of 30 s to compute the 18 hand-crafted features for each axis of the sensor channel.

**Table 7:** Results of the classification of hunger and satiety using RF classifier based on the best features selected with feature importance ranking.

No. of Best Features	Acc. Hungry	Acc. Satiety	Acc	AF1 Score
**18**	**79.65**	**96.08**	**93.43**	**87.86**
54	66.02	94.14	90.00	80.08
72	68.18	95.42	92.00	81.80
90	68.00	94.67	90.86	81.33
108	67.33	94.49	90.57	80.91

Acc: Accuracy; AF1 Score: Averaged macro F1 score.

**Table 8:** Results of the classification of hunger and satiety using feature learning approaches.

Classifier	Acc. Hungry	Acc. Satiety	Acc	AF1 Score
MLP	77.79	81.35	80.14	79.57
**CNN**	**81.37**	**83.70**	**82.90**	**82.54**

Acc: Accuracy; CNN: Convolutional Neural Network; MLP: Multi-Layer Perceptron.

**Figure A1:**
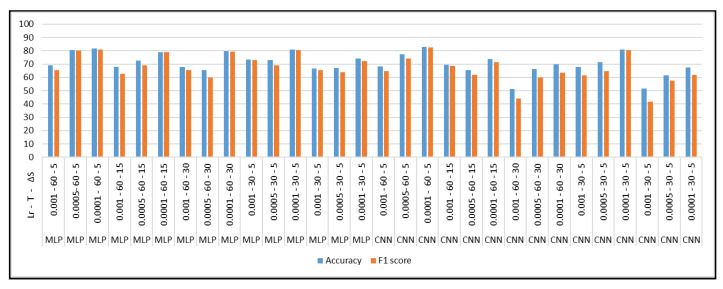
Selection of hyper-parameters for the feature learning approaches. lr: learning rate; T: window size; Δ*S*: step size.

## Data Availability

Data sharing is not applicable to this article.

## Abbreviations

The following abbreviations are used in this manuscript:

VAS	Visual Analog Scales
ANN	Artificial Neural Networks
DNN	Deep Neural Networks
GSR	Galvanic Skin Response
EDA	Electrodermal Activity
EEG	Electroencephalography
fMRI	Functional Magnetic Resonance Imaging
DC	Degree of Centrality
ReHo	Regional Homogeneity
fALFF	Fractional Amplitude of Low Frequency Fluctuations
SF	Spectral Features
CDF	Cepstral Domain Features
GCC	Gammatone Cepstral Coefficients
CFS	Correlation-based Feature Selection
ECG	Electrocardiogram
EMG	Electromyography
PPG	Photoplethysmogram
TMP	Thermopile
EOG	Electrooculography
ML	Machine Learning
BVP	Blood Volume Pulse
MLP	Multi-layer Perceptrons
LSTM	Long Short-term Memory
CNN	Convolutional Neural Network
ReLU	Rectified Linear Unit
SVM	Support Vector Machine
DT	Decision Tree
RF	Random Forest
LOSO	Leave-one-subject-out
ADAM	Adaptive Moment Estimation
SWS	Sliding Window Segmentation
Acc	Accuracy
AF1	Averaged macro F1 score

## Appendix A. Comparison of Manual Feature Selection Approaches

Feature selection (FS) is a process usually applied in machine learning studies that involve the computation of a large number of features. In particular, it is required to eliminate features that would not be the most discriminative for the classification problem to solve, and on the other hand, identify the most useful ones. We used in our study three commonly used FS methods: Boruta, eXtreme Gradient Boosting (XGB), and RF [65,66,67].

RF is an ensemble learner that works well with nonlinear data, handles large datasets efficiently, and is useful for feature selection. Most of the time, it provides better accuracy compared to other algorithms. However, RF can be slow in training when used with a large number of trees, and is sometimes not suitable for many sparse features [48,49,50,53,65].

Similar to RF, XGB is an ensemble machine learning algorithm that incorporates loss minimization using gradient descent to the RF framework. It is less prone to overfitting, can handle missing values, has minimal effects of outliers, and can also be used as a feature selector. However, it is more difficult to tune because there are many hyperparameters and overfitting is possible if the parameters are not set correctly [66,68].

Boruta is a wrapper feature selection approach based on RF that selects or eliminates features after computing an feature importance scores, so that the quality of its feature selection depends on the quality of the RF model. The sensitivity of Boruta can be improved by using a RF with a larger number of decision trees. However, increasing the number of trees in RF may increase the computation time of the Boruta algorithm, which limits the use of the algorithm for analyzing very large datasets [67].

In order to make a fair comparison between the manual FS approaches in this study, we selected the best 18, 54, 72, 90, and 108 features with Boruta, XGB, and RF, and classified them with XGB and RF classifiers. The best results of each classifier in each setting are shown in Table A1. The best configuration was obtained by using RF both for feature selection and classification.

**Table A1:** Results of the classification of hunger and satiety using RF and XGB classifier based on the best features selected with Boruta, XGB, and RF.

Classifier	FS Algorithm	No. of Best Features	Acc. Hungry	Acc. Satiety	Acc	AF1 Score
**RF**	**RF**	**18**	**79.65**	**96.08**	**93.43**	**87.86**
RF	Boruta	108	72.53	95.89	92.86	84.21
RF	XGB	54	73.12	95.88	92.86	84.50
XGB	RF	18	69.23	94.63	90.86	81.93
XGB	Boruta	54	53.33	93.11	88.00	73.22
XGB	XGB	18	63.92	94.20	90.00	79.06

RF: Random Forest; XGB: eXtreme Gradient Boosting; Acc: Accuracy; AF1 Score: Averaged macro F1 score.

## Appendix B. Hyper-Parameter Selection for Feature Learning Approaches

Machine learning algorithms work with two types of parameters, namely learnable parameters and hyper-parameters. The learnable parameters are those that the algorithms learn themselves during training on a given dataset, while hyper-parameters are specified by engineers or scientists prior to the training in order to regulate how algorithms learn, and to change the performance of the model. In our study, the most impactful hyper-parameters on the final classification performances were the learning rate *(lr)*, window size *(T)*, and step size *(*ΔS).

The *lr* determines the rate at which the ANN training algorithm (backpropagation algorithm) updates the weights of the network during each training iteration. More specifically, each neural weight $w_{n}$ at iteration $n\in N^{*}$ is updated following the formula:

$$w_{n}=w_{n-1}-lr\times \frac{\partial L}{\partial w}\left(w_{n-1}\right)$$

where *L* designates the loss function comparing the network outputs to the expected outputs.

The window size *T* and step size Δ*S* are both segmentation parameters that respectively determine how long in time the input of the network is, and how much time needs to pass between two consecutive windows of data. Both parameters control the rate at which the learning algorithm picks up new information.

Figure A1 shows the ANN performances obtained for the various combinations of hyper-parameters that were tested for the feature learning approaches (MLP and CNN) in this study. Since no automated method for optimizing the hyper-parameters of deep neural networks has proven its effectiveness in practice so far, the best values for these parameters in this study were determined through trial-and-error. The hyper-parameter *T* = 60 s, Δ*S* = 5 s, and *lr* = $10^{-4}$ worked best for the MLP and CNN models of this study.

## Appendix C. Demographic Information about the Subjects

The following Table A2 shows the demographic data (such as sex, age, and weight) of the subjects used for data acquisition in this study.

**Table A2:** Demographic data of subjects used for data acquisition in this study.

Subject Name	Sex/Gender	Age (in years)	Weight (in kg)
S1	Female	23	65
S2	Male	29	71
S3	Male	37	72
S4	Male	26	81
S5	Male	27	75

kg: Kilograms; S1: Subject 1; S2: Subject 2; S3: Subject 3; S4: Subject 4; S5: Subject 5.
